# Depletion of regulatory T cells increases T cell brain infiltration, reactive astrogliosis, and interferon-γ gene expression in acute experimental traumatic brain injury

**DOI:** 10.1186/s12974-019-1550-0

**Published:** 2019-08-05

**Authors:** Tobias J. Krämer, Nathalia Hack, Till J. Brühl, Lutz Menzel, Regina Hummel, Eva-Verena Griemert, Matthias Klein, Serge C. Thal, Tobias Bopp, Michael K. E. Schäfer

**Affiliations:** 1 Department of Anesthesiology, University Medical Center of the Johannes Gutenberg-University Mainz, Langenbeckstr. 1 (Bld. 505), 55131 Mainz, Germany; 2 Institute for Immunology, University Medical Center of the Johannes Gutenberg-University Mainz, Langenbeckstrasse 1, 55131 Mainz, Germany; 3 Research Center for Immunotherapy (FZI), Johannes Gutenberg-University Mainz, Mainz, Germany; 4 Focus Program Translational Neurosciences (FTN), Johannes Gutenberg-University Mainz, Mainz, Germany

**Keywords:** Traumatic brain injury, Inflammation, Cytokines, Immune response, T cells, Astrocytes, Microglia

## Abstract

**Background:**

Traumatic brain injury (TBI) is a major cause of death and disability. T cells were shown to infiltrate the brain during the first days after injury and to exacerbate tissue damage. The objective of this study was to investigate the hitherto unresolved role of immunosuppressive, regulatory T cells (Tregs) in experimental TBI.

**Methods:**

“Depletion of regulatory T cell” (DEREG) and wild type (WT) C57Bl/6 mice, treated with diphtheria toxin (DTx) to deplete Tregs or to serve as control, were subjected to the controlled cortical impact (CCI) model of TBI. Neurological and motor deficits were examined until 5 days post-injury (dpi). At the 5 dpi endpoint, (immuno-) histological, protein, and gene expression analyses were carried out to evaluate the consequences of Tregs depletion. Comparison of parametric or non-parametric data between two groups was done using Student’s *t* test or the Mann-Whitney *U* test. For multiple comparisons, *p* values were calculated by one-way or two-way ANOVA followed by specific post hoc tests.

**Results:**

The overall neurological outcome at 5 dpi was not different between DEREG and WT mice but more severe motor deficits occurred transiently at 1 dpi in DEREG mice. DEREG and WT mice did not differ in the extent of brain damage, blood-brain barrier (BBB) disruption, or neuronal excitotoxicity, as examined by lesion volumetry, immunoglobulin G (IgG) extravasation, or calpain-generated αII-spectrin breakdown products (SBDPs), respectively. In contrast, increased protein levels of glial fibrillary acidic protein (GFAP) and GFAP+ astrocytes in the ipsilesional brain tissue indicated exaggerated reactive astrogliosis in DEREG mice. T cell counts following anti-CD3 immunohistochemistry and gene expression analyses of *Cd247* (CD3 subunit zeta) and *Cd8a* (CD8a) further indicated an increased number of T cells infiltrating the brain injury sites of DEREG mice compared to WT. These changes coincided with increased gene expression of pro-inflammatory interferon-γ (*Ifng*) in DEREG mice compared to WT in the injured brain.

**Conclusions:**

The results show that the depletion of Tregs attenuates T cell brain infiltration, reactive astrogliosis, interferon-γ gene expression, and transiently motor deficits in murine acute traumatic brain injury.

## Background

Traumatic brain injury (TBI) is a leading cause of death and disability worldwide and represents a critical public health and socio-economic problem [1]. TBI can range from mild concussions with short-term and reversible symptoms to severe and permanent brain damage. Current treatment options are restricted to surgical intervention and supportive care. However, secondary pathogenic processes, such as disintegration of the blood-brain barrier (BBB), disturbed autoregulation of cerebral blood flow, impaired tissue oxygenation, and mitochondrial metabolism, which result in the expansion of the damage into the surrounding healthy tissue [2–4], are potential targets of therapeutic approaches in TBI [5, 6]. In addition, TBI elicits a robust immune response within hours and days [7]. This immune response is characterized by the infiltration of the damaged tissue by peripheral immune cells and the activation of brain resident astrocytes and microglia, both in patients and in animal models of TBI [8–14]. However, the inflammatory reaction after TBI evokes beneficial and detrimental effects which are still incompletely understood at the mechanistic and molecular level [15–18].

Animal studies using histology, flow cytometry, and/or intravital microscopy provided evidence that neutrophils are the earliest peripheral immune cells entering the injured brain followed by macrophages, dendritic cells, and T cells, eventually after the secondary brain damage occurred [19–22]. T cells infiltrating the damaged tissue were observed at posttraumatic days 3–5 in the controlled cortical impact (CCI) model of TBI [13, 23]. However, reduced numbers of infiltrating T cells were found at posttraumatic day 7 [13, 24]. More recently, persistent chronic T cell infiltration was reported 3 months after lateral fluid percussion injury which, however, did not correlate with the severity of chronic inflammation [25]. Thus, it has been demonstrated that T cells infiltrate the brain parenchyma in TBI during acute and chronic phases. Moreover, some studies indicated that T cells aggravate brain injury [26, 27]. Accordingly, effector CD4+ T cells adoptively transferred into T cell- and B cell-deficient recombination-activating gene 1 (RAG1) knockout mice exacerbated lesion size and apoptosis after brain injury [28]. Experimental studies in the early phase of TBI further indicated that the number of circulating T cells correlated with T cell infiltration and inflammatory responses as well as cell death beneath the impact site [29].

To date, only scarce data exist on the role of regulatory T cells (Tregs) in TBI. This T cell subtype suppresses and thereby controls effector T cells to prevent exaggerated immune responses and in particular autoimmunity [30–32]. Notably, it has been reported that the number of circulating Tregs are positively correlated with a favorable clinical outcome after TBI [33]. Nevertheless, controversial results on the role of Tregs were reported in different types of CNS injuries [34], e.g., either removal or addition of Tregs impaired retinal ganglion cell survival following optic nerve injury [35]. The role of Tregs is also a matter of debate in stroke and its animal models [36–38], which share many pathophysiological features with TBI [39].

In summary, the inflammatory response in TBI is spatially and temporally dynamic and the role of Tregs therein is largely unexplored. Here, we studied this issue in experimental TBI using diphtheria toxin (DTx)-mediated depletion of Tregs in transgenic DEREG mice*.* We subjected mice to the CCI model of TBI, examined neurological and motor deficits until 5 days post-injury (dpi) which corresponds to the acute phase of TBI. The consequences of Tregs depletion were evaluated using behavioral, (immuno-) histological, protein, and gene expression analyses.

## Methods

### Animals and DTx administration

The study was conducted in accordance with the national guidelines, approved by the animal protection committees (Landesuntersuchungsamt RLP, G14-1-026). Adult male mice, 8–10 weeks old, were used. C57Bl/6 DEREG-FoxP3-GFP reporter mice were provided by Lahl et al. [40] and background-matched C57Bl/6 WT mice were purchased (Charles River Laboratories, Sulzfeld, Germany). Group sizes (*n* = 12, each genotype) were calculated prior to approval with the analysis of variance sample size. Animals were housed in compliance with institutional guidelines (Johannes Gutenberg-University Mainz, Germany). All efforts were made to minimize the number of animals and their suffering. Depletion of Tregs was performed in DEREG-FoxP3-GFP mice using intraperitoneal administration of 1 μg of DTx (Merck) 24 h before and 24 h after CCI. C57Bl/6 WT mice were treated identically. Investigators were blind to the genotype groups during all experiments and analyses.

### Flow cytometry

Flow cytometry was used to control for DTx-mediated Treg depletion. Cells were collected from tail vein blood and processed to cell surface marker staining and flow cytometry. To this end, cells were stained with an antibody to CD4 (clone GK1.5, Biolegend) and expressions of CD4 (PE) and FOXP3 (GFP) were analyzed on a FACS LSR II using the DIVA (Becton, Dickinson Bioscience) and FlowJo software (FlowJO, LLC).

### CCI surgery

CCI was performed essentially as described [41, 42]. Briefly, animals were anesthetized with 4 vol% isoflurane inhalation. Rectal temperature was maintained at 37 °C with a feedback-controlled heating pad (Hugo Sachs, March-Hugstetten, Germany). After midline incision and craniotomy, CCI was induced with a custom fabricated impactor (L. Kopacz, Germany; tip diameter, 3 mm; impact velocity, 8 m/sec; impact duration, 150 msec; impact depth, 1 mm). The craniotomy and the skin were carefully closed and the animals transferred to a neonatal incubator (IC8000, Draeger, Luebeck, Germany) for 1.5 h with controlled air temperature (35 °C) and ambient humidity (35%).

### Assessment of neurological impairment and motor deficits

Neurological impairment was assessed at 1 h before CCI, then daily from 1 day to 5 days after CCI using a neurological severity score (NSS) modified from [43] as described [44]. After NSS assessment, mice were examined at 1 day and 5 days after CCI using the rotarod performance test as described [45]. Briefly, four rotarod tests were performed before injury and the average of these trials was taken as the baseline. Following injury, animals were tested in two trials per investigated time point. Post-injury scores from these trials were averaged and evaluated relative to their pre-injury latencies to control for variability in pre-injury performance.

### Histology and immunohistochemistry

Animals were deeply anesthetized with isoflurane, sacrificed by cervical dislocation, decapitated, brains carefully dissected, and processed for histology and immunohistochemistry essentially as described [46]. Brain lesion volume was calculated from 16 consecutive cresyl violet stained sections by summation of areas multiplied by the distance between sections. Data were expressed in percent relative to the volume of the ipsilesional hemisphere. Immunohistochemistry was performed according to standard methods as described [44, 47] using rabbit anti-CD3 (clone SP7, dilution 1:500, Abcam), mouse anti-NeuN (clone A60, dilution 1:500, Chemicon), rabbit anti-Iba1 (dilution 1:1500, WAKO Chemicals), rat anti-GFAP (clone 2.2B10, dilution 1:500, Thermo Fisher), and appropriate secondary biotin- (dilution 1:1,000, Vector laboratories) or AlexaFluor-conjugated antibodies (dilution 1:500, Life Technologies). Images of cresyl violet staining were captured using a stereomicroscope (STEMI 305, Zeiss), CD3/NeuN co-immunostaining and Iba1 immunostaining using an AxioVert200 light microscope equipped with AxioCam (Zeiss). Co-immunostaining of CD3/GFAP were imaged using a confocal microscope (LSM510, Zeiss) and T cells were counted in perilesional brain parenchyma locations in two brain sections for each mouse (bregma − 0.7 mm and − 1.2 mm) using a 40× objective by an investigator blind to the genotype. Images of GFAP immunostaining were captured with a 20× objective and identical acquisition parameters and processed for quantification using ImageJ (NIH Image) with appropriate threshold settings for background subtraction and cell counts using the Analyze Particles plugin (10–1.000 μm^2^).

### Immunoblotting and dot blot immunoassay

Sodium dodecyl sulfate-polyacrylamide gel electrophoresis (SDS-PAGE), immunoblotting, and dot blot immunoassay were performed as described [47, 48]. Briefly, tissue samples were lysed in buffer [50 mM Tris-HCl, pH 7.5; 150 mM NaCl; 1 mM EDTA; 1% NP-40; 0.1% SDS, protease inhibitors (Roche)] and resolved by SDS-PAGE (37.5 μg/lane) or spotted onto nitrocellulose membranes (10 μg/dot), respectively. Primary antibodies specific to αII-spectrin (AA6, dilution 1:1.000, Enzo Life Sciences), GFAP (6F2, dilution 1:2.000, Agilent Dako), Iba1 (dilution 1:500, WAKO Chemicals), and GAPDH (6C5, dilution 1:2.000, Acris) followed by secondary antibodies conjugated to infrared fluorescence IRDyes (dilution 1:15.000, LI-COR Biotechnology) were used. Protein bands and their optical signal intensities were revealed using an Odyssey near-infrared laser imager, measured with Image Studio Version 3.1 software (both LI-COR Biotechnology), and normalized to the sample’s GAPDH signal intensity. Data were expressed as the ratio of the optical signal intensities.

### Gene expression analyses by quantitative real-time PCR

Coronal brain tissue slices were cut at the level of the cortical impact and separated between the left and right hemispheres. Upper right quadrants containing the lesioned and the perilesional brain tissue were snap-frozen in liquid nitrogen, stored at − 80 °C, and further processed for quantitative real-time PCR (qRT-PCR)-based gene expression analyses as described before [47, 49]. Briefly, all assays were carried out in our laboratory by an investigator blind to group allocation. Using specific oligonucleotide primer pairs and optimized temperature conditions for qPCR, values were normalized to the reference gene PPIA (*cyclophilin A*) and absolute quantification was performed using a target-specific standard curve of mRNA copies [49]. The expression data analysis was performed with the LightCycler Software, Version 4.5 (F. Hoffmann-La Roche AG; Basel, CH). Sequences of applied oligonucleotide primer pairs (5′-3′), annealing temperature, amplicon size in base pairs (bp): CD3zeta (*Cd247*, 58 °C, 150 bp): fw-CTG CTA CTT GCT AGA TGG AAT CC, rev-TCT CTT CGC CCT AGA TTG AGC; CD8 (*Cd8a*, 58 °C, 149 bp): fw-GTG GCT CAG TGA AGG GGA C, rev- GGG ACA TTT GCA AAC ACG CT; *IFN-γ* (*Ifng*, 58 °C, 229 bp): fw-GCT CTG AGA CAA TGA ACG CT, rev-AAA GAG ATA ATC TGG CTC TGC; IL-10 (*Il10*, 58 °C, 420 bp): fw-TGT GTC AGC CCT CAG AGT AC, rev-CAC TGA CAC TTC GCA CAA; IL-1β (*Ilb*, 55 °C, 348 bp): fw-GTG CTG TCG GAC CCA TAT GAG, rev-CAG GAA GAC AGG CTT GTG CTC; IL-6 (*Il6*, 55 °C, 471 bp): fw-TCG TGG AAA TGA GAA AAG AGT TG, rev-TAT GCT TAG GCA TAA CGC ACT AG; MHCII HLA-DR gamma (*Cd74*, 58 °C, 84 bp): fw*-*CCG CCT AGA CAA GCT GAC C, rev-ACA GGT TTG GCA GAT TTC GGA; TNFα (*Tnfa*, 62 °C, 212 bp): fw-TCT CAG TTC TAT GGC CC, rev-GGG AGT AGA CAA GGT ACA AC; PPIA (*cyclophilin A*, 58 °C, 146 bp): fw-GCG TCT SCT TCG AGC TGT T, rev-RAA GTC ACC CTG GCA.

### Statistics

Data are expressed as mean ± standard error of mean (SEM). Statistical analyses were analyzed using GraphPad Prism software (La Jolla, California, USA). Data distribution was tested by Shapiro-Wilks test and the comparison of parametric and non-parametric data between two groups was done using Student’s *t* test and the Mann-Whitney *U* test, respectively. For multiple comparisons, *p* values were calculated by one-way ANOVA followed by Tukey’s post hoc test and by Kruskal-Wallis followed by Dunn’s post hoc test for parametric and non-parametric data, respectively. Differences between genotypes over the survival time of 5 days in body weight, NSS, and rotarod performance were calculated using two-way ANOVA followed by Sidak’s multiple comparison. All data sets were tested for statistically significant outliers using the Grubbs’ test. Differences were considered significant when *p* < 0.05.

## Results

### CD3+ T cells infiltrate the injured brain tissue in acute experimental TBI

**Fig. 1:**
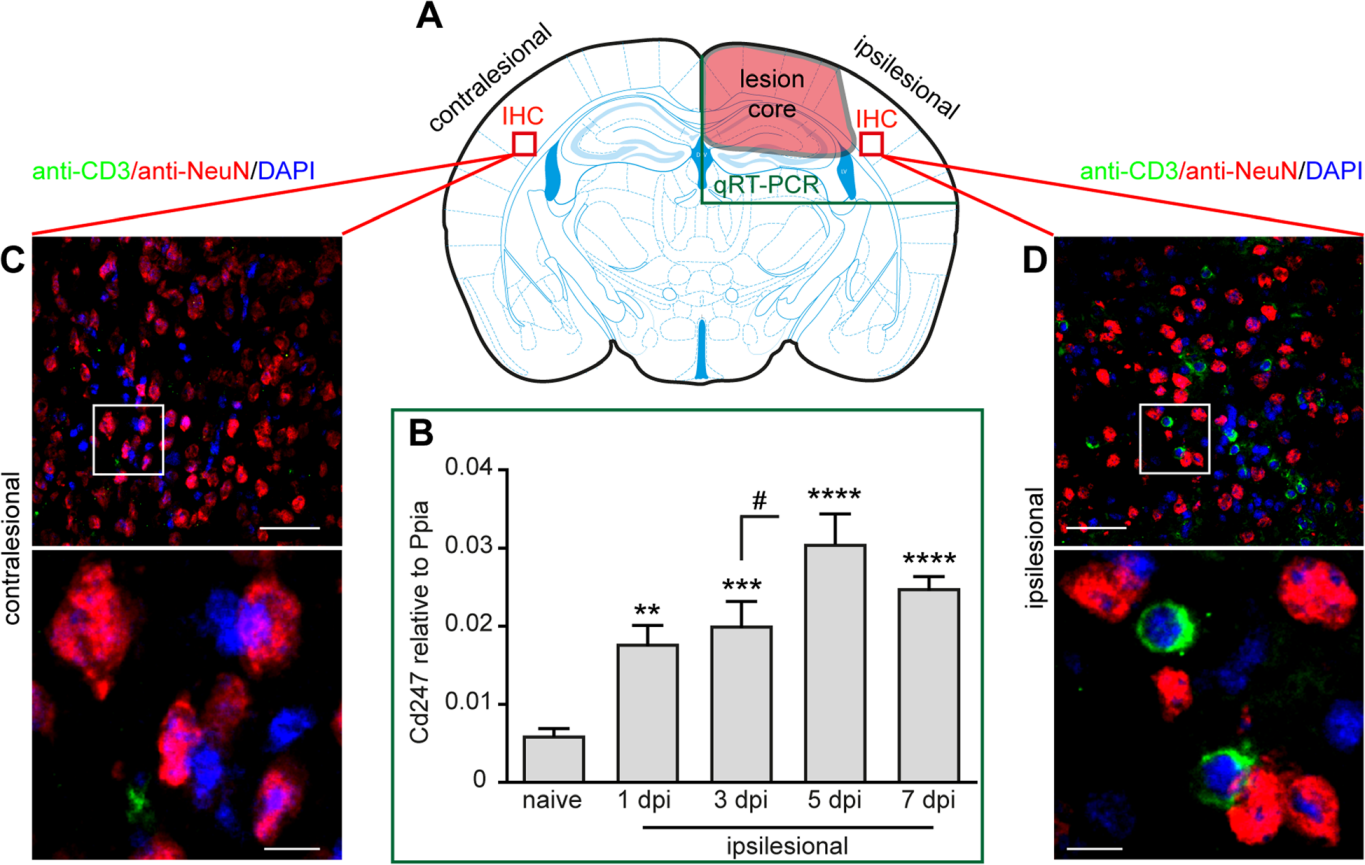
CD3+ T cells infiltrate the injured brain tissue in acute experimental TBI. **a** Scheme illustrating the brain tissue regions examined by qRT-PCR (green box, compared to corresponding regions of naive brains) or immunohistochemistry (IHC, red boxes). **b** qRT-PCR time course analysis of *Cd247* expression in the injured, ipsilesional brain tissue reveals peak expression at 5 dpi. **c**, **d** Double-immunostaining using anti-CD3 (green, pan T cell marker) and anti-NeuN (red, pan neuron marker), and DAPI staining (blue, nuclei). **c** CD3+ T cells were absent in the non-injured, contralesional hemisphere. **d** CD3+ T cells infiltrated the injured, ipsilesional brain tissue. Brain sections from five mice were examined by IHC at 5 dpi. Data are expressed as mean ± SEM (*n* = 9–10 per time point) and statistical significance was calculated by one-way ANOVA followed by Dunn’s multiple comparison test (**p < 0.01; ***p < 0.001; ****p < 0.0001; ^#^*p* < 0.05). Scales: 100 μm, 20 μm (**c**, **d**)

We and others have previously reported that CD3+ T cells infiltrated the injured brain parenchyma at 3–5 days after CCI [13, 23], suggesting their involvement in pathogenic processes. To initially assess the degree of T cell infiltration in the injured brain, we determined CD3zeta (*Cd247*) gene expression as a marker of T cells using qRT-PCR [48] and performed immunohistochemistry (IHC) using pan-antibodies specific to T cells (anti-CD3) or neurons (anti-NeuN) (Fig. 1a–d). Determination of *Cd247* mRNA expression in ipsilesional compared to naive brain tissues indicated that T cell infiltration increased from 1 dpi to 7 dpi and reached a peak at 5 dpi. Furthermore, *Cd247* mRNA expression was significantly increased from 3 dpi to 5 dpi (Fig. 1b). Qualitative assessment of anti-CD3 immunostaining demonstrated that T cells were absent in the non-injured, contralesional brain parenchyma (Fig. 1c) but present in the injured, ipsilesional brain parenchyma at 5 dpi (Fig. 1d). These results suggested that injury-induced T cell infiltration proceeds during the first days after CCI and is restricted to injury sites.

### DTx-mediated depletion of Tregs in DEREG mice

**Fig. 2:**
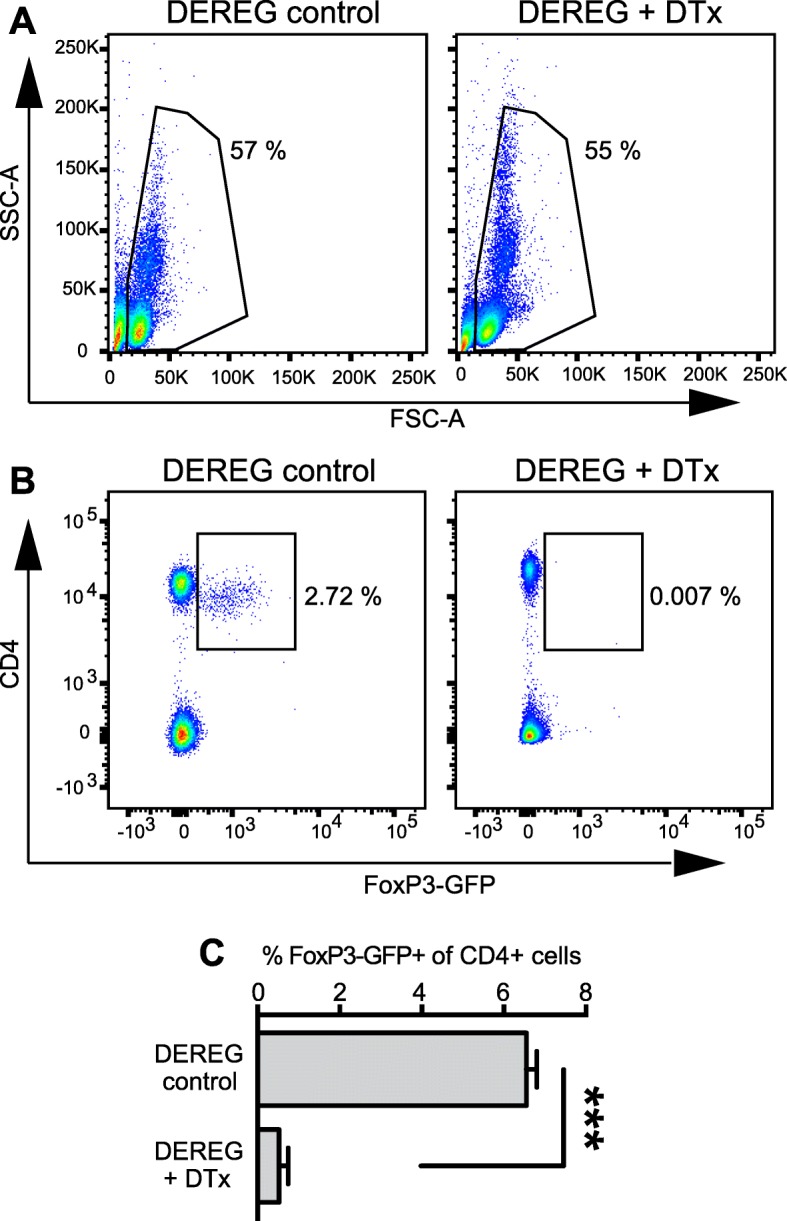
DTx-mediated depletion of Tregs in DEREG mice. **a** FACS plot examples of blood lymphocyte gates from naive DEREG mice (control) or DTx-treated DEREG mice showing the lymphocyte populations for naive DEREG mice (control) and for DEREG + DTx at 5 days after the last DTx administration. **b** FACS plot examples showing CD4+ lymphocytes vs. FoxP3-GFP+ Tregs in blood samples from DEREG control mice and DEREG DTx-treated mice. **c** Histogram showing depletion of FoxP3-GFP+ Tregs in DTx-treated DEREG mice. Data represent mean ± SEM (*n* = 5–10) and statistical significance was calculated by Mann-Whitney *U* test (***p < 0.001)

Tregs suppress and thereby control T cells to prevent exaggerated immune responses in various diseases and organs. In order to investigate the role of Tregs and T cells in experimental TBI, we took advantage of transgenic DEREG-FoxP3-GFP mice (DEREG) to deplete Tregs that express the human diphtheria toxin DTx receptor [40, 50]. Depletion was done according to Lahl et al. [40] using two injections (i.v., 1 μg DTx each, the second injection 48 h after the first injection). The total number CD4+ FoxP3-GFP+ Tregs in blood samples was almost abolished at day 5 after the second DTx injection (Fig. 2a–c). The numbers of other lymphocyte subsets were not affected in this model, as reported before [40].

### Treg depletion does not affect the overall neurological outcome but transiently aggravated motor deficits after CCI

**Fig. 3:**
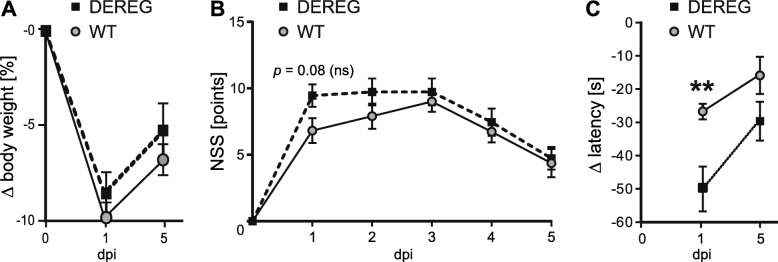
Treg depletion did not affect the overall neurological outcome but transiently aggravated motor deficits after CCI. **a**–**c** Body weight, neurological impairment (NSS), and rotarod performance of DEREG and WT mice (*n* = 11–12, each genotype). Body weight and rotarod performance are expressed as *∆* relative to pre-injury values (set to 0). **a** Relative body weight loss at 1 dpi and 5 dpi was similar between DEREG and WT mice. **b** NSS at 1–5 dpi were not significantly altered between DEREG and WT mice but DEREG mice showed a trend towards an increased NSS at 1 dpi (*p* = 0.08). **c** Motor deficits assessed by rotarod performance were transiently aggravated in DEREG at 1 dpi but not at 5 dpi compared to WT mice. Data are expressed as mean ± SEM. Statistical significance between DEREG and WT mice was calculated using two-way ANOVA followed by Sidak’s multiple comparison test (**p < 0.01)

We subjected DEREG and WT C57Bl/6 mice to the CCI model of TBI using identical dosage and injection time points for DTx (24 h before and 24 h after CCI, 1 μg of DTx each). The survival time was set to 5 dpi, the aforementioned peak time point of *Cd247* expression in the ipsilesional brain tissue (Fig. 1). The two groups of mice were monitored for body weight and neurological impairments using a composite NSS [44], and the motor performance was assessed in the rotarod task (Fig. 3a–c). Initial body weight loss at 1 dpi and its partial recovery at 5 dpi were similar between DEREG and WT mice (Fig. 3a). CCI led to pronounced neurological deficits throughout the observation period from 1 dpi to 5 dpi. A recovery period from 3 dpi to 5 dpi was evident both in DEREG mice and WT mice. DEREG mice showed a trend towards an increased NSS at 1 dpi (DEREG 1 dpi, 9.46 ± 0.86; WT 1 dpi, 6.58 ± 0.89; *p* = 0.08) but the NSS was similar compared to WT at 5 dpi (DEREG 5 dpi, 4.73 ± 0.85; WT 5 dpi, 4.33 ± 0.98; Fig. 3b). However, DEREG mice showed a reduced rotarod performance at 1 dpi but not at 5 dpi compared to WT mice (Fig. 3c; DEREG 1 dpi, − 50.05 ± 6.79; WT 1 dpi, − 26.86 ± 2.14, *p* = 0.019; DEREG 5 dpi, − 29.64 ± 5.84; WT 5 dpi, − 16.63 ± 5.17, *p* = 0.30). Thus, Treg depletion did not affect the overall neurological outcome but transiently aggravated motor deficits after CCI.

### DEREG mice exhibit no alterations in brain lesion size and IgG extravasation compared to WT mice after CCI

**Fig. 4:**
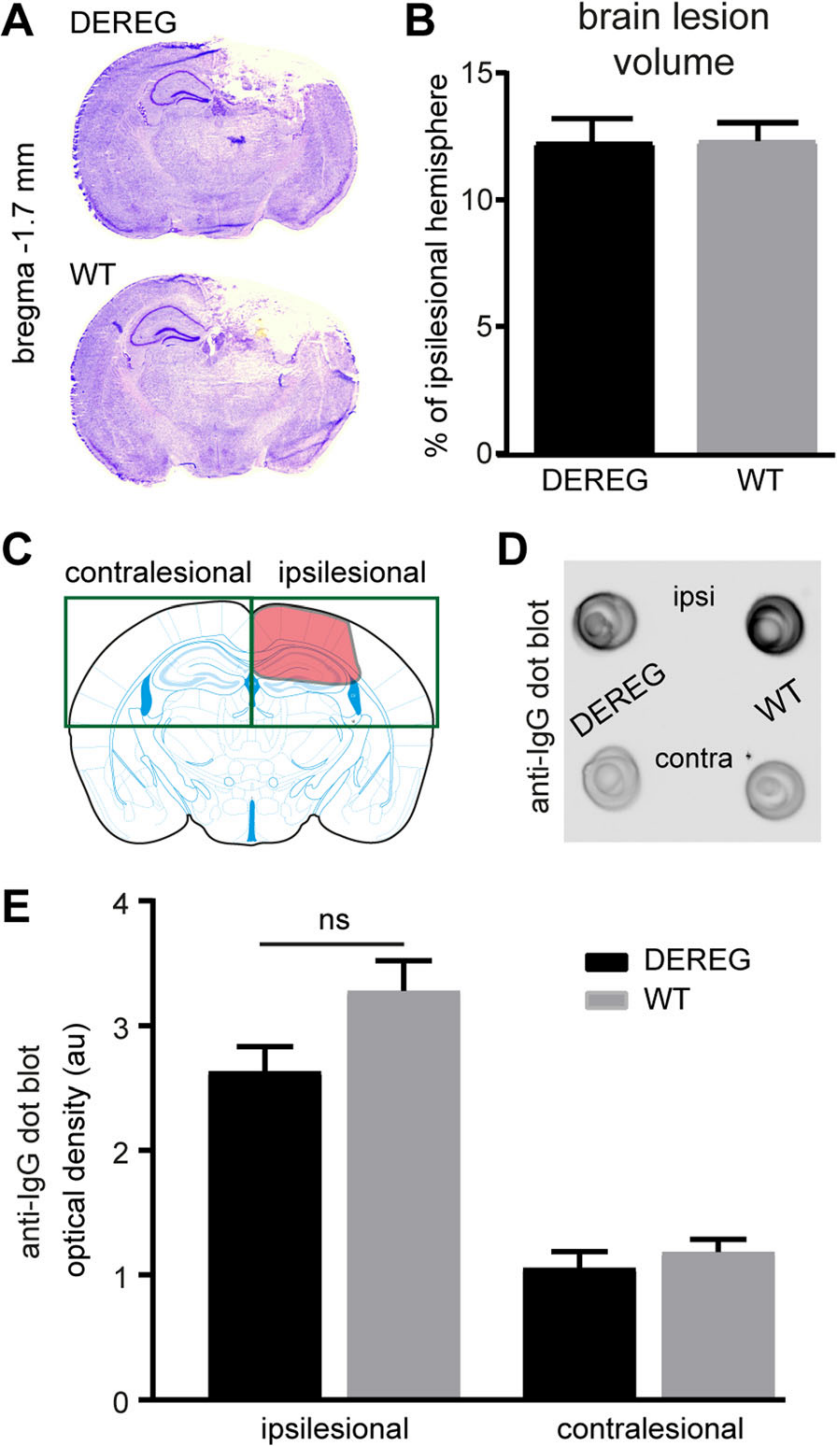
DEREG mice exhibit no alterations in brain lesion size and IgG extravasation compared to WT mice after CCI. **a** Examples of cresyl violet-stained cryosections demonstrating the extent of unilateral brain damage at 5 dpi. **b** Brain lesion volume (percentage of ipsilesional hemisphere) was not altered between DEREG and WT mice (*n* = 11–12, Mann-Whitney *U* test). **c** Scheme illustrating the brain tissue regions collected for anti-IgG dot-blot immunoassay. **d** Example of dot-blot immunoassay using samples from ipsi- or contralesional brain tissues and probed with antibodies specific to mouse IgG. **e** IgG levels in ipsilesional brain tissues from DEREG and WT mice were not significantly (ns) different (Fig. 4e, *p* = 0.063, one-way ANOVA followed by Tukey’s multiple comparison test)

During the first days after the primary injury, TBI triggers secondary processes such as edema formation, BBB disruption, and Ca^2+^ excitotoxicity which lead to progressive loss of vital brain tissue [2]. To determine the extent of brain lesions at 5 dpi, consecutive brain cryosections were stained with cresyl violet (Fig. 4a). Brain lesion volumetry revealed a similar extent of the brain lesions in DEREG and WT mice (Fig. 4b, DEREG 12.2 ± 0.9%; WT 12.3 ± 0.7%). We next determined the amount of IgG in the ipsi- or contralesional brain tissues (Fig. 4c) as a proxy for CCI-induced BBB impairment using dot blot immunoassay [47]. We found strongly increased ipsilesional vs. contralesional IgG levels both in DEREG and WT mice at 5 dpi (Fig. 4d) and a trend towards reduced ipsilesional IgG levels in DEREG mice which, however, did not reach a statistically significant level (*p* = 0.063, Fig. 4e). Taken together, brain lesion size and IgG extravasation were not different between DEREG and WT mice after CCI.

### DEREG mice show similar induction of neuronal injury and microgliosis marker but elevated protein levels of the reactive astrocyte marker GFAP

**Fig. 5:**
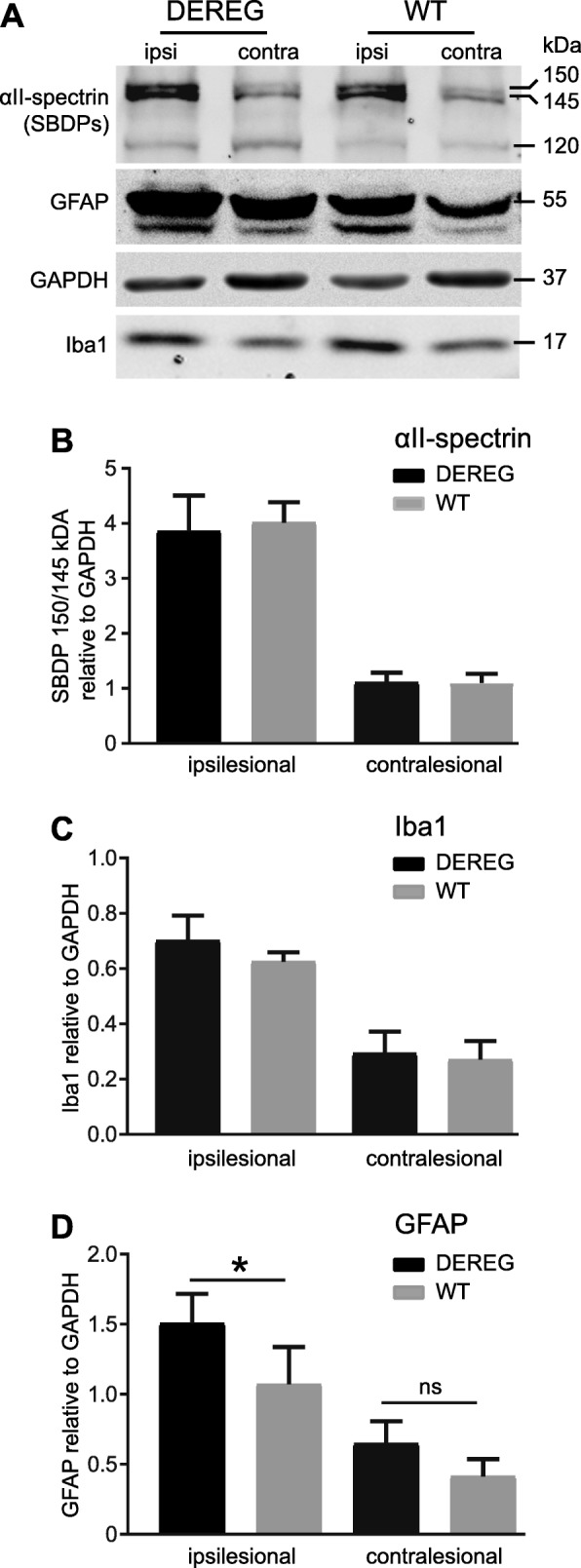
DEREG mice show similar induction of neuronal injury and microgliosis marker but elevated protein levels of the reactive astrocyte marker GFAP. **a** Western blot of brain lysates (37.5 μg protein/sample) from ipsi- and contralesional brain tissue. Blots were probed with antibodies specific to αII-spectrin, Iba1, GFAP, or GAPDH. **b** CCI induces the generation of αII-spectrin breakdown products of 150 kDa, and 145 kDa in the ipsilesional brain tissue. The 120 kDa αII-spectrin fragment was not altered by CCI. No differences were found between DTx-treated DEREG mice compared to WT. **c** Protein expression of the microglia marker Iba1 appeared increased in the ipsilesional brain tissue after CCI. There were no alterations between DEREG and WT mice. **d** GFAP was induced by CCI in the ipsilesional brain tissue. Increased GFAP protein levels were found in DEREG mice compared to WT. Data are expressed as mean ± SEM, *n* = 11 per group, *p < 0.05, one-way ANOVA followed by Tukey’s multiple comparison test

We next examined the CCI-induced and calpain-mediated generation of 145/150 kDa αII-spectrin breakdown products (SBDPs) which can be utilized to assess Ca^2+^-mediated excitotoxicity and an overall disturbance of Ca^2+^ homeostasis in TBI [51, 52]. Additionally, we determined protein levels of the microglia/macrophage marker Iba1 and the astrocyte marker GFAP in DEREG and WT mice. Using SDS-PAGE and western blot of protein lysates from ipsi- and contralesional brain regions collected at 5 dpi, we found that CCI strongly increased the amount of SBDPs and the protein levels of Iba1 and GFAP in samples from ipsi- compared to contralesional brain tissues, both in DEREG and WT mice (Fig. 5a–d). SBDPs and Iba1 protein levels did not differ significantly between DEREG and WT mice (Fig. 5b, c). In contrast, the ipsilesional protein levels of GFAP were significantly elevated in tissue samples from DEREG mice compared to WT (Fig. 5d, DEREG ipsi 1.50 ± 0.21; WT ipsi 0.86 ± 0.17; *p* = 0.042).

### Treg depletion causes increased reactive astrogliosis after CCI

**Fig. 6:**
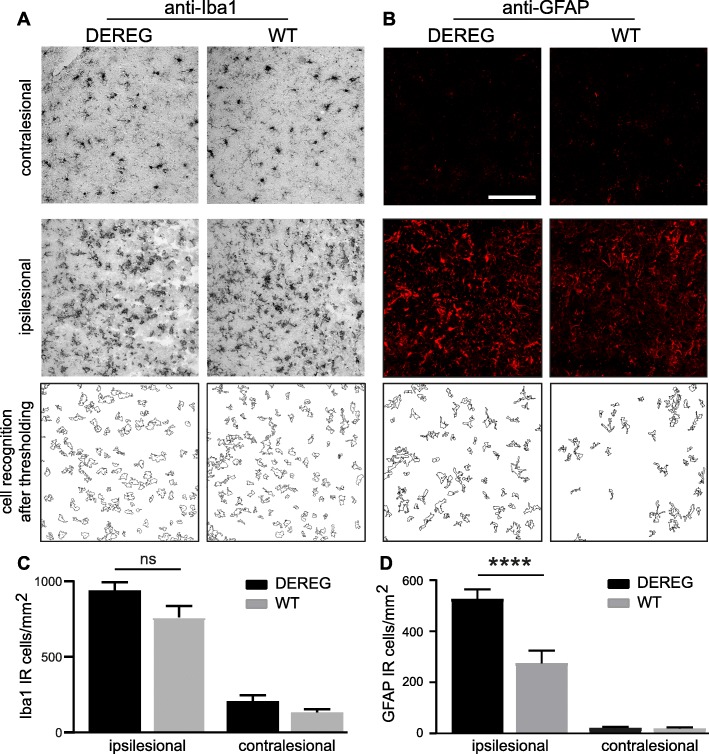
DEREG mice develop exaggerated astrogliosis after CCI. **a**, **b** Images showing details of brain cryosections after immunostaining with anti-Iba1 (**a**) or anti-GFAP (**b**) to label microglia/macrophages or reactive astrocytes in the contralesional or ipsilesional cortex at 5 dpi, respectively. Images were processed for digital cell recognition after thresholding. **c**, **d** Histograms showing the numbers of Iba1 IR cells or GFAP IR cells per mm^2^. GFAP IR cells were markedly increased in ipsilesional brain parenchyma of DEREG compared to WT mice. Data are expressed as mean ± SEM, *n* = 11 per group, ****p < 0.0001, one-way ANOVA followed by Tukey’s multiple comparison test. Scale 100 μm (**b**)

Data from western blot analyses suggested increased reactive astrogliosis in DEREG mice after CCI. To substantiate these findings, brain sections were immunostained using anti-Iba1 or anti-GFAP to reveal the number of microglia/macrophages or astrocytes at 5 dpi, respectively (Fig. 6a, b). Following immunohistochemistry, microscope images were acquired in perilesional regions in the injured, ipsilesional hemisphere and in corresponding regions of the non-injured, contralesional hemisphere (IHC, Fig. 1a). Iba1 immunostaining revealed marked microgliosis in the ipsilesional hemisphere around the injury both in DEREG and WT mice while the number of contralesional Iba1 immunoreactive (IR) cells was lower (Fig. 6c). The mean number of ipsilesional or contralesional Iba1 IR cells was not significantly different between DEREG and WT mice (Fig. 6c).

GFAP IR was scarce in the contralesional hemisphere but strong GFAP IR was observed in the ipsilesional hemisphere around the injury, both in DEREG and WT mice (Fig. 6b). However, cell counts demonstrated that DEREG mice displayed a markedly increased number of GFAP IR astrocytes in the ipsilesional region of interest compared to WT (Fig. 6d; DEREG ipsi 526.2 ± 38.2; WT ipsi 274.7 ± 49.5; *p* < 0.0001). These results indicate that the depletion of Tregs causes increased reactive astrogliosis after CCI.

### T cell infiltration of the injured brain tissue is increased in DEREG mice

**Fig. 7:**
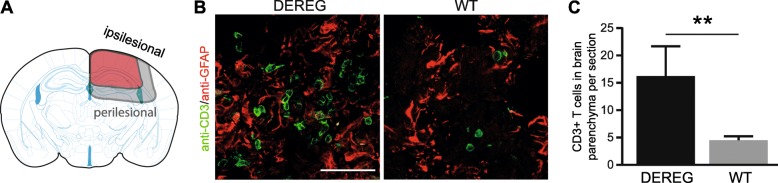
T cell infiltration of the injured brain tissue is increased in DEREG mice. **a** Scheme illustrating the perilesional regions (gray) containing infiltrations of CD3 IR T cells. **b** Double-immunofluorescence images of cryosections showing CD3 IR T cells (green) infiltrating the injured perilesional brain parenchyma as indicated by the presence of GFAP IR astrocytes (red) at 5 dpi. **c** Histogram showing the mean number of CD3 IR cells infiltrating the brain parenchyma per brain section. Statistical significance was calculated by Mann-Whitney *U* test (**p < 0.01). Scale 100 μm (**b**)

To reveal possible consequences of Treg depletion on the number of T cells infiltrating the injured brain, we performed immunohistochemistry using antibodies specific to CD3 or GFAP to identify T cells and reactive astrocytes. Close inspection of double-immunostained brain sections revealed a pattern of isolated and clustered CD3+ T cells located in the perilesional brain parenchyma in the presence of GFAP+ reactive astrocytes (Fig. 7a, b). Clusters of CD3+ T cells were completely absent in the brain parenchyma of the contralesional tissue and only very few isolated cells were occasionally observed in a minority of animals and brain sections (data not shown). The mean number of CD3+ T cells per section found to infiltrate the perilesional brain parenchyma was low; however, the number of CD3+ T cells was strongly increased in DEREG compared to WT mice (Fig. 7c, DEREG ipsi 16.2 ± 5.4; WT ipsi 4.5 ± 2.5; *p* = 0.008). These findings indicate that the depletion of Tregs leads to increased T cell infiltration of the injured brain parenchyma after CCI.

### Treg depletion leads to increased gene expression of T cell markers and IFN-γ after CCI

**Fig. 8:**
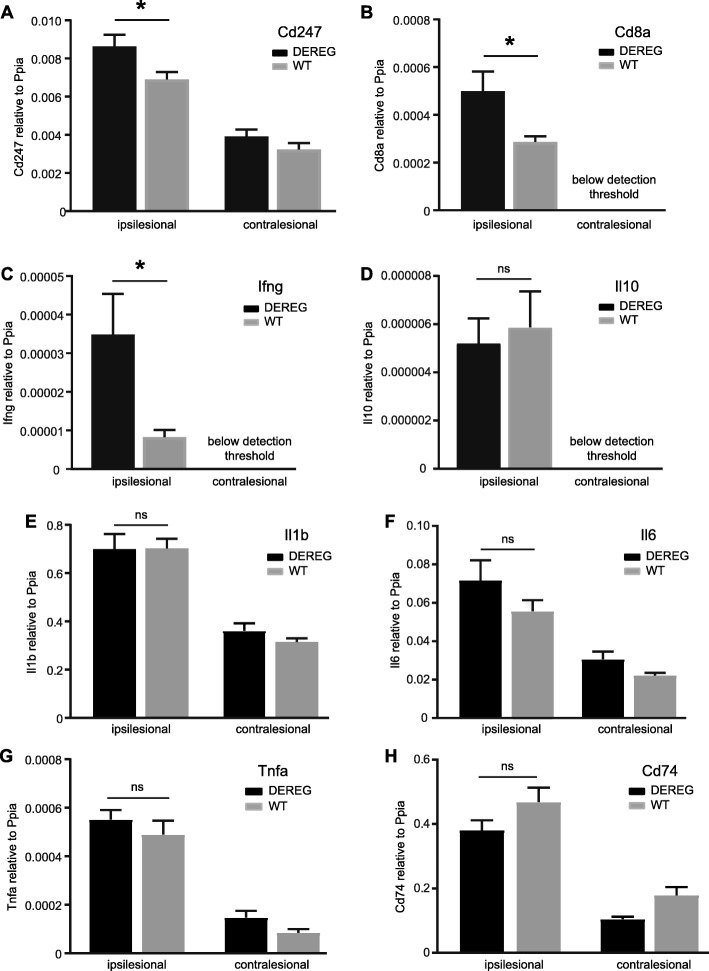
Increased gene expression of T cell marker and IFN-γ in the brain tissue from DEREG mice after CCI. **a**–**h** Histograms showing mRNA expression of T cell marker genes (*Cd247*, *Cd8a*) and inflammatory marker genes (*Ifng*, *Il10*, *Il1b*, *Il-6*, *Tnfa*, and *Cd74*) in WT and DEREG mice as determined by qRT-PCR at 5 dpi. Data from ipsilesional and contralesional brain tissue samples are shown. **b**–**d** Note that *Cd8a*, *Ifng*, and *Il10* gene expression levels in the contralesional brain tissue samples were below the detection threshold. Data are expressed as mean ± SEM, and *p* values were calculated by Student’s *t* test (**b**–**d**; *p < 0.05) or by one-way ANOVA followed by multiple comparison using Tukey’s or Dunn’s post hoc test (**a**, **e**–**h**, *p < 0.05)

We next determined gene expression levels of the T cell markers CD3zeta (*Cd247*) and CD8 (*Cd8a*) and the inflammation markers interferon (IFN)-γ (*Ifng*), IL-10 (*Il10*), IL1β (*Il1b*), IL-6 (*Il6*), TNFα (*Tnfa*), and MHCII (*Cd74*) in ipsi- or contralesional brain samples from DEREG and WT mice at 5 dpi using qRT-PCR (Fig. 8a–h). In agreement with the results from immunohistochemistry, the expression levels of CD3zeta and CD8 mRNA were higher in ipsilesional samples from DEREG compared to WT mice (Fig. 8a, b). Similarly, the gene expression of IFN-γ was significantly increased in ipsilesional samples from DEREG compared to WT mice (Fig. 8c). However, gene expressions of other inflammation markers were strongly induced in the ipsilesional brain samples but not different between DEREG and WT mice (Fig. 8d–h). Thus, the depletion of Tregs leads to increased gene expression of T cell markers and IFN-γ in the ipsilesional brain tissue after CCI.

## Discussion

The objective of this study was to investigate the hitherto unresolved role of Tregs in TBI. We subjected DEREG mice to the CCI model of TBI and examined neurological impairment and motor deficits from 1 dpi to 5 dpi, considered as the acute phase of CCI. Our results from histopathological and neurological analyses at 5 dpi do not support a critical role of Tregs for the extent of structural brain damage or the overall neurological outcome. However, Treg-depleted DEREG mice exhibited a trend towards increased NSS and transiently aggravated motor deficits at 1 dpi and increased reactive astrogliosis in the injured brain hemisphere at 5 dpi. Furthermore, we found an increased number of CD3+ T cells infiltrating the perilesional brain parenchyma and elevated IFN-γ gene expression in DEREG mice indicating an immunomodulatory role of Tregs in the brain response to traumatic injury.

The extent of the structural brain damage and the amount of SBDPs as a proxy of Ca^2+^-dependent neuronal excitotoxicity [51] were not different between DEREG and WT mice. These findings indicate that Tregs play a minor role for neuronal survival in the acute phase of TBI. These findings are consistent with results of a genetic approach of Treg depletion after experimental cerebral focal ischemia in mice; Tregs alone, respectively Foxp3 expressing cells, were not capable to limit brain damage or improve functional outcome in mice [53]. On the other hand, depletion of Tregs with anti-CD25 mAb profoundly increased delayed brain damage and deteriorated functional outcome [54]. However, anti-CD25 mAb depletes all CD25+ cells, which includes precursor, mature, and activated T and B cells [55] which may contribute to conflicting results from different approaches of Treg depletion in the MCAO model of stroke.

In the present study, we found overall increased GFAP protein levels in lysates from injured brain tissue of DEREG mice and an increased number of GFAP+ astrocytes indicating exaggerated reactive astrogliosis. Reactive astrogliosis is a general response after brain lesion, which has been linked both to beneficial and detrimental processes [56]. Normally, astrogliosis and scar formation improve the outcome and provide neuronal protection, BBB repair, and restriction of CNS inflammation [57], but astrocytes can also adopt a neurotoxic phenotype at injury sites [58]. Furthermore, astrocytes become reactive in response to neuroinflammatory stimuli and peripheral immune cells can communicate with astrocytes and other brain resident cells to create a highly inflammatory microenvironment [18]. However, these processes depend on different contexts, e.g., astrocytes may recruit T cells into the brain parenchyma at early stages while helping to clear T cells from the brain in later inflammation resolution [59]. On the other side, T cells (i.e., Th17 effectors) were proposed to preferentially target astrocytes to promote neuroinflammation [60]. Together with our results showing that DEREG mice are harboring more CD3+ T cells and GFAP+ astrocytes at perilesional sites, these findings suggest a T cell-dependent mechanism leading to preferential activation of astrocytes and reactive astrogliosis. In support of this hypothesis, gene expression of IFN-γ, a major T cell-released factor in the immunological activation of astrocytes [61, 62], was significantly increased in DEREG mice after CCI.

Intriguingly, IFN-γ is associated with greater disability in the post-acute phase after severe TBI in humans [63] and can modulate neuronal networks [64]. Other studies associated impaired motor function with increased expression of inflammation-related genes either in response to environmental factors [65] or to genetic factors [66]. Along with the observation of increased IFN-γ expression, our results from the rotarod performance test indicate that the depletion of Tregs causes transient aggravation of motor deficits at 1 dpi. In addition, a trend towards increased neurological deficits was observed at 1 dpi in DEREG mice. These effects are likely due to the DTx-mediated depletion of Tregs, as previous investigations confirmed that untreated DEREG do not show phenotypic abnormalities compared to C57Bl6 mice [67]. Possibly, the rotarod task may represent a more sensitive task to assess motor impairment in the context of murine TBI. It has been reported that rotarod performance is a very sensitive and efficient index for assessing motor impairment compared to the beam balance and beam walking tasks [68], which are parts of the composite NSS used in the present study [44]. However, neither the rotarod task nor the NSS revealed differences between DEREG and WT mice at 5 dpi, the endpoint of our study. Thus, our findings point to a (immuno-) modulatory role of Tregs without major impact on brain tissue loss and persistent neurological impairment in TBI. A major limitation of the present study is that only a single posttraumatic time point has been investigated. To better understand the immunomodulatory role of Tregs in TBI, additional studies are required including long-term studies to examine their contribution to chronic brain inflammation and associated co-morbidities such as epilepsy and neuropsychiatric disorders. Along this line, a recent study demonstrated that Tregs during the chronic phase of experimental stroke suppress reactive astrogliosis and enhance neurological recovery [69]. Also, molecular characterization of immune cell infiltrations and reactive astrocytes in the injured brain using cell sorting and single-cell RNA sequencing applications in combination with appropriate animal models might be instrumental to reveal molecular pathways and targets for preclinical studies. Furthermore, adoptive transfer of Tregs after TBI may exert neuroprotective and anti-inflammatory actions similar to previous findings in animal models of post-stroke hemorrhage [70] or intracerebral hemorrhage using autologous blood infusion [71]. It will be further important to investigate whether adoptive transfer of Tregs may slow or prevent effector T cell brain infiltration and their potentially detrimental actions in animal models of stroke and TBI.

Up to date, inflammation targeted therapy has not been translated into the clinical setting of TBI, despite promising results in animals studies. Previous attempts to directly influence T cells in humans resulted in a life-threatening systemic inflammatory response syndrome, leading to multiorgan failure after administration of an anti-CD28 monoclonal antibody [72]. In clinical practice, self-tolerance is especially important in patients with autoimmune diseases or after organ transplantation and immunosuppressive drugs such as rapamycin are widely used. Experimental research provided evidence that rapamycin decreases the number of CD4+ T cells and simultaneously increases CD4+/CD25+ Tregs [73, 74]. Also in murine cerebral ischemia, Treg amplification with a CD28 superagonistic monoclonal antibody [75] or rapamycin treatment [76] attenuated the neurological outcome after stroke induction. These effects are to some extent in line with our conclusions and are most probably caused by the modulation of Tregs. In support of this hypothesis, an increased number of Tregs in the circulation appears to be associated with a better outcome after TBI in humans [33]. However, recent work suggests that conclusions on the numerical increase of Tregs are complicated due to the fluctuating expression of Foxp3 and CD25 and therefore may not be decisive for the therapeutic success by modulation of Tregs [77].

## Conclusions

This study suggests a pathophysiological role of Tregs in the CCI model of TBI. Our results show that systemic depletion of immunosuppressive Tregs leads to increased T cell infiltration of the injured brain parenchyma. The presence of T cells in the brain coincides with enhanced IFN-γ gene expression and exaggerated reactive astrogliosis. Thus, depletion of Tregs attenuates acute immune responses in the brain and Tregs may serve a critical function in modulating the pathophysiology of TBI.

## Abbreviations

BBB: Blood-brain barrier
CCI: Controlled cortical impact
DEREG: Depletion of regulatory T cell
Dpi: Days post-injury
Dtx: Diphtheria toxin
FACS: Fluorescence-activated cell sorting
GFAP: Glial fibrillary acidic protein
Iba1: Ionized calcium-binding adapter molecule 1
Ifng: Interferon-γ
IgG: Immunoglobulin G
IL-10, IL1β, IL-6: Interleukin-10,interleukin-β1, interleukin-6
MCAO: Middle cerebral artery occlusion
MHCII: Major histocompatibility complex class II
NSS: Neurological severity score
SBDP: Spectrin breakdown product
TBI: Traumatic brain injury
TNFα: Tumor necrosis factor-α
Treg: Regulatory T cell
WT: Wild type
