# Correction to: Depletion of regulatory T cells increases T cell brain infiltration, reactive astrogliosis, and interferon-γ gene expression in acute experimental traumatic brain injury

**DOI:** 10.1186/s12974-019-1577-2

**Published:** 2019-09-07

**Authors:** Tobias J. Krämer, Nathalia Hack, Till J. Brühl, Lutz Menzel, Regina Hummel, Eva-Verena Griemert, Matthias Klein, Serge C. Thal, Tobias Bopp, Michael K. E. Schäfer

**Affiliations:** 1 Department of Anesthesiology, University Medical Center of the Johannes, Gutenberg-University Mainz, Langenbeckstr. 1 (Bld. 505), 55131 Mainz, Germany; 2 Institute for Immunology, University Medical Center of the Johannes Gutenberg-University Mainz, Langenbeckstrasse 1, 55131 Mainz, Germany; 3 Research Center for Immunotherapy (FZI), Johannes, Gutenberg-University Mainz, Mainz, Germany; 4 Focus Program Translational Neurosciences (FTN), Johannes Gutenberg-University Mainz, Mainz, Germany

**Correction to: J Neuroinflammation (2019) 16:163**

**https://doi.org/10.1186/s12974-019-1550-0**

Following publication of the original article [1], the authors opted to correct the following mistakes. According to the title and our results, the conclusions in the abstract and at the end of the discussion the term “attenuates” must be corrected to read as “increases”.

1. Conclusions (Abstract): “The results show that the depletion of Tregs increases T cell brain infiltration, reactive astrogliosis, interferon-γ gene expression, and transiently motor deficits in murine acute traumatic brain injury”.
2. Conclusions (Discussion): “Thus, depletion of Tregs increases acute immune responses in the brain and Tregs may serve a critical function in modulating the pathophysiology of TBI”.

The authors apologize for the inconvenience caused.
